# Special presentation of bronchobiliary fistula after transcatheter arterial chemoembolization: A case report

**DOI:** 10.1097/MD.0000000000031596

**Published:** 2022-11-18

**Authors:** Yuan-Chun Lo, Ping-Wen Hsu, Fatt-Yang Chew, Hung-Yao Chen

**Affiliations:** a Department of Medical Education, Chang Gung Memorial Hospital, Taoyuan, Taiwan; b School of Medicine, China Medical University, Taichung, Taiwan; c Department of Medical Imaging, China Medical University Hospital, Taiwan; d Center for Digestive Medicine, Department of Internal Medicine, China Medical University Hospital, Taichung, Taiwan.

**Keywords:** biloma, bronchobiliary fistula, hepatocellular carcinoma, TACE, transcatheter arterial chemoembolization

## Abstract

Transcatheter arterial chemoembolization (TACE) is a widely adopted treatment for advanced stage hepatocellular carcinoma (HCC). Nevertheless, several complications may occur, such as hepatic artery injury, nontarget embolization, pulmonary embolism, hepatic abscess, biloma, biliary strictures, and hepatic failure. However, bronchobiliary fistula is rarely mentioned before.

**Patient concerns:**

A 65-year-old man with HCC underwent the TACE procedure, and then he encountered fever, dyspnea, abdominal pain, and abundant yellowish purulent bronchorrhea.

**Diagnosis:**

Bronchobiliary fistula was diagnosed based on the computed tomography (CT) scan of his chest, which revealed the right lower lobe of his lung was connected to a hepatic cystic lesion.

**Interventions:**

Percutaneous transhepatic cystic drainage was performed, and we obtained yellowish bile, showing the same characteristics as the patient’s bronchorrhea.

**Outcomes:**

We kept drainage of his biloma and provided supportive care as the patient wished. Unfortunately, the patient passed away due to progressive right lower lobe pneumonia 2 weeks later.

**Lessons:**

This case exhibits a typical CT scan image that was helpful for the diagnosis of post-TACE bronchobiliary fistula. Post-TACE bronchobiliary fistula formation hypothesis includes biliary tree injuries with subsequent biloma formation and diaphragmatic injuries. Moreover, the treatment of bronchobiliary fistula should be prompt to cease pneumonia progression. Therefore, we introduce this rare complication of post-TACE bronchobiliary fistula in hopes that future clinicians will keep earlier intervention in mind.

## 1. Introduction

Liver cancer is a worldwide disease and has been estimated to exceed 1 million cases by 2025.^[1]^ Among these, hepatocellular carcinoma (HCC), which is highly related to the infection of hepatitis B virus and hepatitis C virus, is the most common type of liver cancer. Transcatheter arterial chemoembolization (TACE) has been widely used as a curative treatment for intermediate-stage HCC, which are unsuitable for resection.^[1]^ However, despite its significant anti-tumor effects, the TACE procedure may induce several complications, such as hepatic artery injury, nontarget embolization, pulmonary embolism, hepatic abscess, biliary strictures, hepatic failure, and intrahepatic biloma formation.^[2,3]^ Previous research demonstrated that conservative percutaneous drainage could manage biloma and have a good prognosis in most cases. In this case report, we share a rare complication, bronchobiliary fistula related to TACE-induced biloma that failed with conservative drainage.

## 2. Case report

A 65-year-old man with chronic hepatitis C was diagnosed with hepatitis C-induced HCC. Complete staging revealed AJCC staging cT4N1M0, Barcelona clinic liver cancer stage C. He went through 6 cycles of TACE and received two courses of palliative radiation therapy for HCC in the S4 and S6 of the liver in the past 15 months. Besides, he took Sorafenib (Nexavar^®^) as a systemic treatment. Computed tomography (CT) scan 2 months after the last TACE demonstrated a cystic lesion with surrounding daughter cysts without ring enhancement developed in S7, where the previous HCC was located. Thus, biloma was highly suspected, but no symptoms and signs were noted at regular follow-up. A month later, he encountered sudden onset of dyspnea accompanied by fever and yellowish sputum for a day. As a result, he sought medical help at another hospital, where he was treated as liver abscess and pneumonia. Percutaneous abscess drainage was placed. The bacterial culture from drainage revealed Citrobacter koseri. Piperacillin/tazobactam and meropenem were prescribed for 5 days. However, there were no significant clinical improvements, except for his fever. Subsequently, the patient was transferred to our hospital.

**Figure 1.**
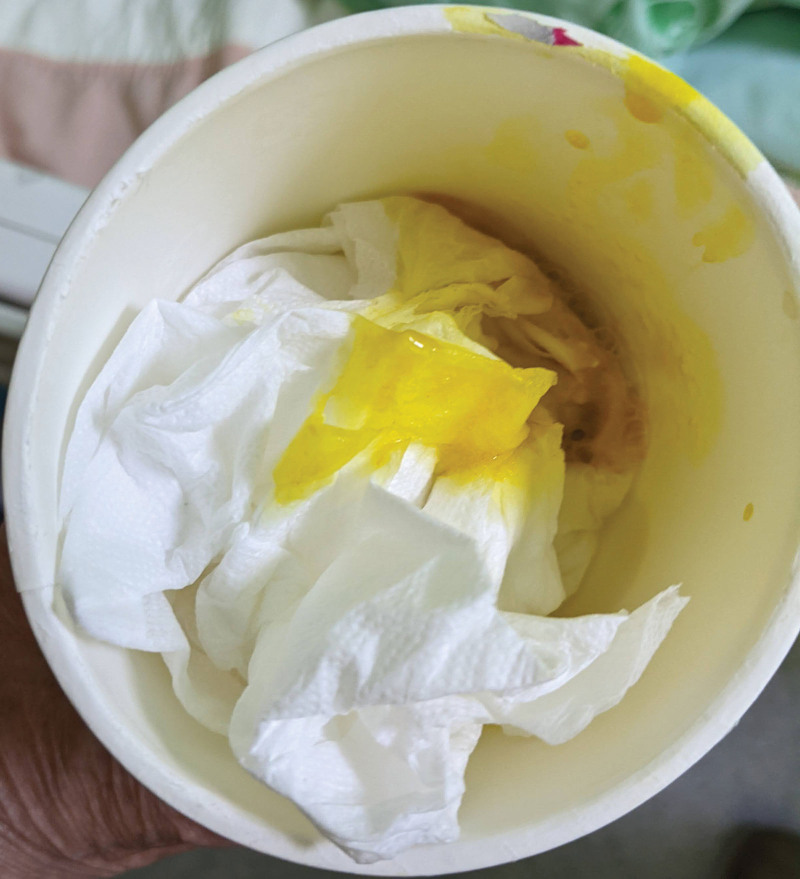
Yellowish bronchorrhea and yellowish nasal discharge (bilioptysis) were recorded during the hospital course.

**Figure 2.**
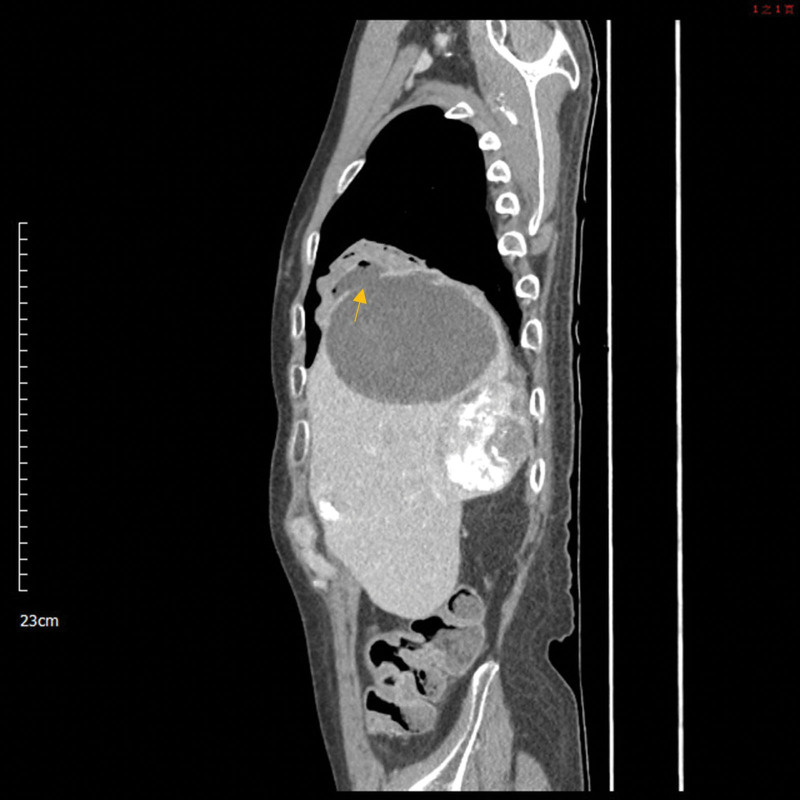
CT scan revealed a biloma and bronchial cyst with an air-fluid level connecting through a diaphragmatic defect (arrow). In addition, an inflammatory pulmonary consolidation in the lateral basal segment of the right lower lobe was also visible. CT = computed tomography.

At our hospital, he presented dyspnea, abundant yellowish purulent bronchorrhea, and yellowish nasal discharge (Fig. 1). Symptoms of severe right upper quadrant abdominal pain (Visual Analogue Scale: 5–6), jaundice and decreased breath sounds with rales in the right lower lung had also been noted. The differential blood count revealed leukocytosis with the left shift. Blood levels of hsCRP and total bilirubin exceeded the reference range, 19.42 mg/dL, and 2.33 mg/dL, respectively. A sputum smear showed no leukocytes or bacteria. His chest CT scan showed that the right lower lobe of his lung was connected to a hepatic cystic lesion and was filled with fluid (Fig. 2). We placed an 8 Fr ring catheter percutaneous drainage for the hepatic cystic lesion and obtained yellowish bile with the same characteristics as the patient’s bronchorrhea. According to the chest CT images and the presentation of cough with bilioptysis, the diagnosis of biloma with bronchobiliary fistula was confirmed. Four weeks later, the followed-up CT scan showed shrinkage of biloma. However, due to persisted bilioptysis, we suggested surgical ligation for the curative treatment of bronchobiliary fistula (BBF). Unfortunately, left hemianopsia developed, and the brain magnetic resonance imaging showed a right occipital region metastatic tumor with cerebral edema. Furthermore, progression dyspnea with desaturation forced us to cancel surgical intervention. Two weeks later, the patient passed away due to progressive right lower lobe pneumonia.

## 3. Discussion

Biloma means a well-circumscribed bile collection in the extra-biliary regions that is a common complication after TACE. Furthermore, a bronchobiliary fistula is an abnormal channel between the biliary system and bronchial tree through the diaphragm.^[4,5]^ In previous research, the incidence of intrahepatic biloma occurring after TACE was 1.04%.^[2]^ Statistically, the mean interval time between intrahepatic biloma formation and the most recent TACE procedure was 69.1 days.^[2]^ However, the incidence of BBF had rarely been described.^[4,5]^ Cases of BBF formation after TACE-induced biloma have been reported even less. In our case, the biloma was discovered 2 months after TACE, similar to previous reports’ interval time. Furthermore, the subsequent formation of the BBF was around a month later. This rare complication, unfortunately, resulted in severe pneumonia and subsequent death in our case. Therefore, we hope to highlight this unique complication and be aware that BBF may form in a month after biloma was noted.

In a review article, a patient diagnosed with BBF usually bases on clinical symptoms.^[4]^ Initial presentations include bilioptysis, pleural effusion, atelectasis, liver abscess (cyst), and intrahepatic bile duct dilatation.^[4,6]^ Among these, bilioptysis is the most common symptom in almost all these patients.^[6]^ The image finding, the fistulous tract connecting pleural effusion and biliary tracts, was rarely seen on image scans (magnetic resonance imaging or CT) in previous reports, namely 2 out of 11 patients.^[4]^ Other diagnostic interventions include percutaneous cholangiogram, magnetic resonance cholangiopancreatography, 99mTcMebrofeninscintigraphy, sputum examination for bilirubin, and contrast-enhanced CT study can also help.^[7,8]^ In our case, yellowish purulent bronchorrhea (serous in consistency) gave us a high suspicion of bilioptysis. Furthermore, the visible diaphragmatic defect on the CT scan can also confirm the diagnosis. Besides, an air-fluid level in the bronchial cyst proved the connection to the bronchus. The before mentioned are markable features on CT that can support the diagnosis of BBF.

Although the etiology of BBF formation after TACE remains unclear, some explanations exist. Iodized oil or other embolic materials used in the TACE procedure may cause ischemia in the peribiliary capillary plexus.^[2]^ Therefore, the damage to the intrahepatic bile ducts will lead to biloma formation.^[2,9]^ The increased intracavitary pressure in biloma possibly drives bile into the chest.^[4]^ Furthermore, infection, thermal injury by radiofrequency ablation, and surgical damage to the diaphragm provide diaphragmatic injuries that contribute to the development of BBF.^[4,10,11]^ As in our case, even though the TACE procedure did not directly injure the diaphragm, bacteria isolated from the biloma indicated the infection status and may contribute to diaphragmatic injuries that form BBF.

The successful management of BBF is mostly drainage and close monitoring.^[5]^ However, in some cases with conditions such as ongoing bile leaks may require surgical fixation.^[4,6,12,13]^ There is no official guideline for the treatment of bronchobiliary fistula. However, a systematic literature review suggests open surgery should be the first choice after interventional techniques have failed or when BBF is secondary to tumors and biliary obstruction.^[6]^ Returning to our case, the patient underwent the intervention of percutaneous drainage, but it failed to cease the deteriorating condition. Therefore, we consulted a chest surgeon for fistula ligation. Nevertheless, surgical intervention was postponed due to his progressive dyspnea.

In conclusion, despite the slime possibility, the TACE procedure might induce the formation of biloma and may subsequently cause BBF in a patient with a diaphragmic injury. Its unique presentation of bilioptysis and the fistula track on CT scans can be a way to confirm the diagnosis of BBF. This case reminds us to be aware of the possibility of BBF formation after the TACE procedure, which can be a fatal complication. Moreover, patients may benefit from earlier diagnosis and earlier surgical intervention.

## Author contributions

Yuan-Chun Lo and Ping-Wen Hsu contributed to writing the manuscript. Fatt-Yang Chew contributed to image interpretation. Hung-Yao Chen was responsible for patient care and manuscript preparation. All authors read and approved the final manuscript.

**Conceptualization:** Yuan-Chun Lo, Ping-Wen Hsu, Hung-Yao Chen.

**Data curation:** Yuan-Chun Lo, Ping-Wen Hsu.

**Formal analysis:** Yuan-Chun Lo.

**Project administration:** Hung-Yao Chen.

**Software:** Fatt-Yang Chew.

**Supervision:** Hung-Yao Chen.

**Validation:** Ping-Wen Hsu, Fatt-Yang Chew, Hung-Yao Chen.

**Writing – original draft:** Yuan-Chun Lo, Ping-Wen Hsu.

**Writing – review & editing:** Yuan-Chun Lo, Ping-Wen Hsu, Hung-Yao Chen.
